# The pleasure of publishing

**DOI:** 10.7554/eLife.05770

**Published:** 2015-01-06

**Authors:** Vivek Malhotra, Eve Marder

**Affiliations:** Cell and Developmental Biology Program, Center for Genomic Regulation, Barcelona, Spainvivek.malhotra@crg.eu; Department of Biology and the Volen National Center for Complex Systems, Brandeis University, Waltham, United Statesmarder@brandeis.edu

**Keywords:** scientific publishing, publishing, peer review, open access, eLife

## Abstract

When assessing manuscripts eLife editors look for a combination of rigour and insight, along with results and ideas that make other researchers think differently about their subject.

The senior editors at eLife are often asked: ‘Where is the bar for an eLife paper?’ Another frequent question is: ‘Why should I submit my best work to eLife?’ The second of these questions is not surprising because it is human nature to be wary of anything new and challenging. The first question has its origins in our collective experience of trying to publish in journals that became very exclusive in the days when print and distribution costs limited the number of papers and pages that journals could publish. Here we prefer to explain what we think makes a paper suitable for eLife, and how the journal's peer review process works.

For us, the ideal eLife paper presents an accurate description of data that makes others in the field think differently and moves the field forward. An eLife paper should give the reader the pleasure of reading about elegant or clever experiments, of learning something new, of being challenged to think about their subject in a new way, or of seeing a particularly stunning image that has meaning because it shows some of the secrets of life. Our goal at eLife is to publish papers that our reviewers and editors find authoritative, rigorous, insightful, enlightening or just beautiful. Of course, beauty is in the eye of the beholder, and ideas about what is beautiful can change over time. Nonetheless, some things will always be truly beautiful, such as great art and great music, and the same is true for great science. Happily, eLife has no restrictions on how many papers we can publish, or any strictures on how many we should reject. Consequently, our editorial challenge is to recognize excellent papers and to encourage authors, reviewers and editors to divest ourselves of the behaviours that have diminished the pleasure of doing science and publishing the results.

Many eLife editors are old enough to remember the days when we submitted manuscripts by mail. This entailed collating three or four copies of the typescript along with glossy figures that were photographic prints of artwork or raw data. When the package arrived at the editor's office, it was opened, assigned a number, and then sat on the editor's desk, sometimes for weeks if he (and it was almost always he) was out of town. The editor then assigned reviewers, and a copy of the manuscript was mailed to each reviewer (usually without asking whether they were prepared to review the paper because it was assumed that, if asked, one would agree to review). The reviewers would prepare their reports and mail them back to the editor, who would eventually send a decision letter to the author. The whole process often took 2–3 months, sometimes longer, which sounds terrible by today's standards. Nonetheless, when we mailed a paper it was with a sense of joy and accomplishment, coupled with relief because we knew it was off our desks and psyches for enough time to put some emotional and intellectual distance between ourselves and the manuscript.

Even 40 years ago, there were options of where to publish, but each field had one or two flagship journals that were generally considered the ‘best’. These journals were most often published by professional societies and characteristically published authoritative and detailed papers, replete with controls and methods. Indeed, many of those ‘best journals’ commonly published two or three papers in a row from the same laboratory that developed an entire story. It was not an accident that the classic Hodgkin and Huxley papers, which elucidated and modelled the ionic mechanism underlying the action potential, were published as a series of five back-to-back papers totalling 75 figures and 120 pages in the *Journal of Physiology* in 1952. The first paper included a long and detailed description of the equipment and the newly developed voltage-clamp method (with 7 figures devoted to methods). The last paper, with its 23 figures and 44 pages, is perhaps the most famous paper in computational neuroscience, and remains required reading for all students entering the field. But what makes these papers (and many other great papers) remarkable is that they combine the new data with insight into the thinking that led to the new experiments. When we go back to classic papers we understand the mind of the scientist at work: whether the paper was 2 pages or 22 pages, we can see the original logic of the work.

Most reviewers, meanwhile, asked themselves the following questions: ‘are the data interesting?’; ‘does the manuscript make sense?’; and ‘do the data support the author's contentions?’ Some manuscripts were rejected, obviously, but more often the review process was seen as a mechanism to improve the final, published paper. In recent years new journals have sprung forth and the number of papers has grown enormously. Sadly, as editors have been deluged with manuscripts, one negative review can blackball a worthy manuscript. Of course a single thoughtful reviewer may provide a deeper understanding of what is wrong with a paper than a casual positive review, but it is important that editors do not automatically reject manuscripts because they have received one negative review.

The eLife editorial process is designed so that each manuscript is handled by an editor who is able to evaluate the science themself. Moreover, after the last review has been received, each reviewer is asked to comment on the other reviews: for manuscripts that have been favourably reviewed, the aim of this consultation process is to agree what revisions are essential to ensure acceptance of a revised manuscript. The authors then receive a decision letter explaining the revisions that are required, rather than being asked to respond to two or three reviewer reports that may be inconsistent with each other, and possibly even contradictory.

What have we learned after two years of publishing at eLife? The most common complaint from reviewers is that authors are overselling their work. We understand that competition for funding and pages in prestige journals has taught authors to frame their work in the most globally ambitious terms. However, there is a fine line between trying to express in a crisp and compelling manner the contribution made by a manuscript and making claims that are beyond what the manuscript does or could do. Indeed, much of the discussion in our consultation sessions revolves around what a paper actually has achieved and what it hasn't achieved, and much of our effort goes to ensure that all eLife papers accurately describe the experiments done and the data collected.

The most common complaint from reviewers is that authors are overselling their work.

An on-going problem is that reviewers have become accustomed to asking for more experiments. eLife's policy is to respect the authors' vision of what they want their paper to be, and to assume that they have thought hard about how far to take a given story. The job of editors and reviewers is to decide if this vision is publishable or not: it is not the job of the editor or reviewer to define the scope of the paper. In the early days of eLife, a review from a new assistant professor asked the authors to perform a number of substantial new experiments before submitting a revised version. When asked by the eLife editor in charge of the review process if these extra experiments were critical or not, the reviewer answered: ‘I thought my job as a reviewer was to always ask for more experiments, but they aren't really necessary’. It is sad that our younger scientists have only known a world in which it is assumed that reviewers always have to ask for substantial new experiments.

We know that the eLife editorial process isn't perfect. Undoubtedly, we have declined to review some important papers, and have rejected other papers on the basis of reviewer comments that were well-intended but might have missed the point. And we have probably published papers that will turn out to be inconsequential. We are acutely aware that peer review, at its best, is not perfect. That said, we believe our pioneering consultation process improves the review process. On numerous occasions one reviewer has challenged a statement made by another reviewer, and the resulting outcome was more substantive and scientifically correct. Many of our reviewers find that this aspect of the review process brings back the joy of scientific discourse around ideas.

A central feature of eLife is that we are able to publish excellent papers, no matter how many figures they contain: we can also embed raw data and movies in papers. Another feature of eLife is that we are open to papers that challenge received wisdom, as circumstances and data change, and to papers that cross disciplinary boundaries. We also hope that eLife has the perspective to recognize that authors invest a great deal in their work, and that while reviewers may be more ‘objective’ than authors, it is the authors who sign the paper and put their reputations on the line, not the reviewers. Reviewers can help to ensure that papers make sense and that the work presented is rigorous. However, it is authors who provide creativity, imagination, and years of hard work.

As editors we should remember that we have an obligation to authors and readers to publish work that pushes the field forward, and that those authors who entrust their best papers to eLife honour us by so doing. Moreover, we are just as interested in work that improves our understanding of basic biological processes as we are in work that has obvious medical applications. The life and biomedical sciences are changing rapidly, and the importance of quantitative methods is increasing, so we strive to remain flexible enough to handle new developments and fields as they arise, while eschewing the temptations to follow fads.

Our goal is to recapture the best aspects of an era that provided scientists with the space they needed to tell a story properly, while benefitting from the phenomenal opportunities offered by the digital technologies of today and tomorrow.

**Figure 1:**
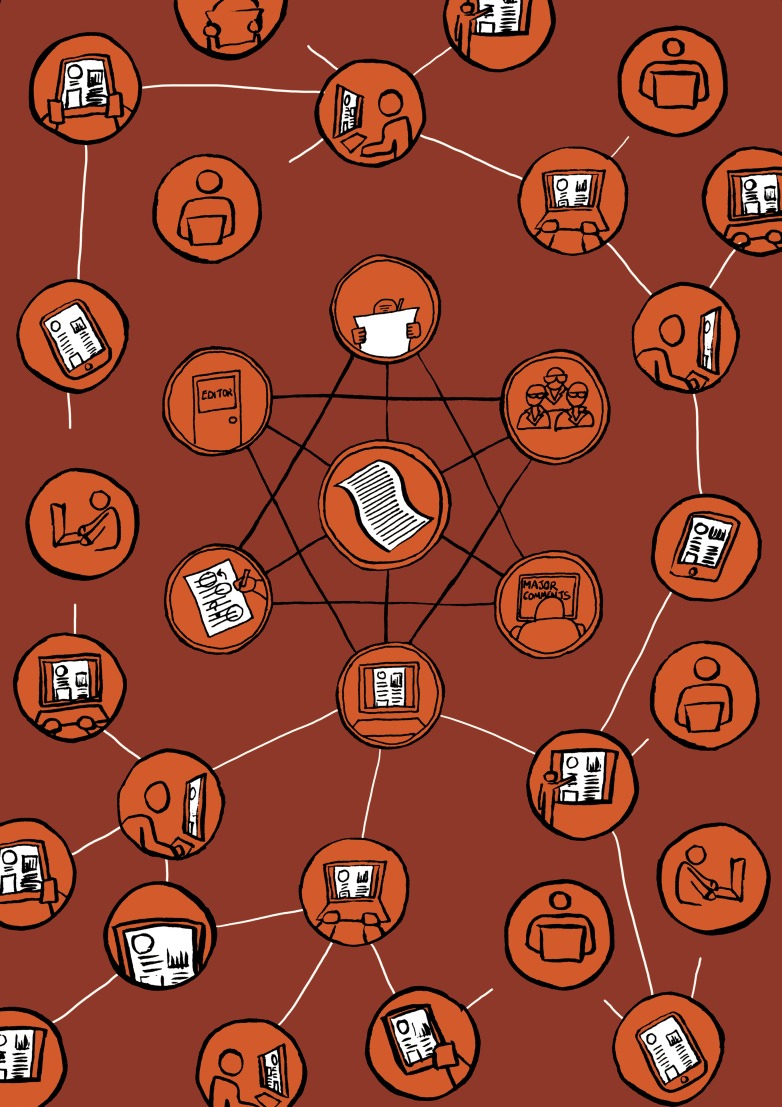
The editors and reviewers at eLife work with authors to publish papers that make other researchers think differently about their subject.

Illustration http://www.claudiastocker.com

